# Identification of Multisystemic Therapy (MST) Subgroups with Distinct Trajectories on Ultimate Outcomes in Norway

**DOI:** 10.1007/s10802-020-00735-3

**Published:** 2021-01-06

**Authors:** Serap Keles, Knut Taraldsen, Asgeir Røyrhus Olseth

**Affiliations:** 1 Norwegian Center for Child Behavioral Development, P.O. Box 7053, 0306 Oslo, Majorstuen Norway; 2 Knowledge Center for Education, University of Stavanger, Stavanger, Norway

**Keywords:** Multisystemic therapy, Quality assurance, Evidence-based practices, Youth problem behavior, Latent class growth analysis, Immigrant status

## Abstract

The effect of Multisystemic Therapy (MST) treatment for serious behavior problems among adolescents has been established through multiple studies. However, variations across individuals should also be examined to better understand how MST works or for whom. In this study, we explored and identified subgroups of youth with serious problems in Norway regarding their responses to MST in terms of ultimate MST outcomes (e.g., living at home, abstaining from violence) over time. We further explored whether immigrant background, in addition to gender and age of the youth at intake, predicted belonging to the subgroups. Data came from 1674 adolescents (*Mean*_Age_ = 14.55, *SD*_*Age*_ = 1.58; 60.7% boys) and their families referred to MST treatment by the municipal Child Welfare Services for serious and persistent antisocial behavior. The outcomes were assessed at five time-points from intake to 18-months after discharge for youth and families who completed the treatment. Latent class growth analyses revealed heterogeneous trajectories regarding youths’ responses to MST. Results indicated a high and sustained degree of improvement across the ultimate outcomes for the vast majority of the youths. However, there was still variation in the groups, with improvement and deterioration trajectories for various outcomes. Most of these trajectories were predicted by gender and youth’s age at intake, but not by immigrant status. Not every youth-at-risk responds similarly to MST, and more studies examining heterogeneity will help us to identify factors to be targeted to better tailor the MST interventions for youth with serious problems.

## Introduction 

The effect of Multisystemic Therapy (MST) treatment for serious behavior problems among adolescents has been established through multiple studies carried out in various countries as well as in meta-analyses (Asscher et al. 2014; Curtis et al. 2004; Henggeler 2011, 2012; Ogden and Halliday‐Boykins 2004; Sundell et al. 2008; Van der Stouwe et al. 2014). MST decreases youth problem behavior including criminal behavior and substance abuse through working directly with families and improving family functioning. However, to our knowledge, except two studies (Halliday-Boykins et al. 2004; Mertens et al. 2017), most of the extant studies evaluated the success of MST in reducing serious problem behavior by examining the average changes in the participants. However, the variations across individuals should also be examined to better understand how MST works or for whom. Halliday-Boykins et al. (Halliday-Boykins et al. 2004), and Mertens et al. (Mertens et al. 2017), on the other hand, examined the trajectories of change in adolescents’ externalizing and/or internalizing behaviors who received MST, and found various subgroups responding differently to treatment. For example, Mertens et al. (Mertens et al. 2017) identified six groups of youth, four of which showed a positive effect of MST with decreases in externalizing behaviors, while two of which displayed poor treatment response by showing either no change or increase in externalizing behaviors. Halliday-Boykins et al. (Halliday-Boykins et al. 2004), on the other hand, identified five trajectories following psychiatric crisis, based on change in externalizing and internalizing symptoms as a result of either MST or psychiatric hospitalization. A substantial proportion of youth representing three trajectories displayed sustained high levels of symptoms, while two trajectories showed a decrease in the symptoms. However, their sample consisted of youth with severe psychiatric symptoms requiring hospitalization, hence not representing the target group of standard MST directed at youth with externalizing problems (Mertens et al. 2017).

In the current study, as a part of continuous quality improvement, we investigated the heterogeneity in how youth with serious problems in Norway did respond to MST in terms of ultimate MST outcomes (i.e., living at home, attending school/work, not in trouble with the law, abstaining from substance abuse, and abstaining from violence) over time. We further explored whether immigrant background, in addition to gender and age of the youth at intake, predicted belonging to the subgroups.

Multisystemic Therapy (MST) is an evidence-based, intensive family- and community-based treatment program targeted at youth aged between 12 and 18 years with serious behavior problems, which may include delinquency and substance abuse (Henggeler et al. 2009; MST Services Inc. 2020). It is based on social-ecological theory (Bronfenbrenner 1979), and addresses known risk and protective factors at the youths’ various social arenas such as home, school, peers, and community. The MST model includes integrated, explicit implementation requirements and strategies, such as directions for organizational support, and routines and systems for evaluation and continuous quality improvement. In Norway, MST has been implemented nationally, guided by the Ministry of Child and Family Welfare, and supported by a national implementation team at The Norwegian Center for Child Behavioral Development (NUBU) tasked with evaluating and maintaining the quality of the implementation.

The MST team and therapist are accessible twenty-four hours a day, seven days a week, and engage the parents and the youth in continuous prioritization of goals, analysis of problem drivers, development and implementation of interventions, and evaluation of outcomes. The treatment goal is to reduce the youth’s problem behavior through empowering parents, increasing family warmth, cohesion, and responsible behavior within and outside the family, hence reducing risk factors for the youth’s further development, averting placement out of home, and setting the youth off on a positive developmental trajectory.

Even though positive outcomes of MST have been found in several studies, the effects might still be moderated by various factors including the characteristics of the youth such as gender, age and immigrant status. While earlier MST studies generally found no moderating effects of gender, age, or ethnicity, researchers are still encouraged to include such variables especially with the move of these programs into communities (Ogden and Hagen 2006).

Research reveals gender related similarities and differences in both manifestations and development of antisocial behavior (see Ogden and Hagen 2009 for detailed discussion). Gender differences in MST outcomes, on the other hand, have not been examined as a central topic in MST efficacy (Ogden and Hagen 2009). Among the limited number of studies, a Norwegian RCT follow-up study (Ogden and Hagen 2006) investigated gender differences in treatment outcomes and showed that generally MST works similarly effective for girls and boys, but is particularly more effective at keeping boys and older ones at home. In another multi-informant study (Ogden and Hagen 2009), gender differences emerged for some treatment outcomes and when reported by some informants. For example, girls showed better results in parent reported externalizing problems and self-reported delinquency, while boys reported higher reduction in internalizing problems at discharge (Ogden and Hagen 2009). On the other hand, teacher ratings revealed equally beneficial outcomes of MST for boys and girls. Hence, gender may emerge as a significant predictor of MST outcomes, for some outcome areas and when reported by some informants.

MST has been argued to be a culturally competent intervention (Brondino et al. 1997). However, cultural differences that families are embedded in, such as culturally appropriate parenting behaviors, youth-parent relations, and acculturation process, may create a variation on how youths and families with immigrant and non-immigrant background engage in and respond to MST (Fox et al. 2017). Van der Stouve and colleagues (Van der Stouwe et al. 2014) conducted a meta-analysis on the effectiveness of MST building on previous meta-analytical reviews (Curtis et al. 2004; Littell 2005), and found a small but significant treatment effect on delinquency, psychopathology, substance use, out-of-home placement and so on. This meta-analysis also examined under which conditions MST was the most effective. While no difference for the effect of MST was found for the proportion of boys across studies, larger effect sizes were demonstrated with an average participants' age of under 15 years and in studies with a larger proportion of Caucasian juveniles (Van der Stouwe et al. 2014). This indicates that MST is more effective with younger and non-ethnic minority youth. With regard to age, possible explanations for effect in favor of younger children was that older children may have a longer history of confrontations with their parents, development of more dysfunctional and coercive interaction patterns in the family, in addition to the increasing academic and behavioral demands in school with older age (Ogden and Hagen 2008). With regard to immigrant background, this meta-analytic effect was inconsistent with that of the primary studies (e.g., Henggeler et al. 2002) testing for moderating effects of ethnicity and reporting no such effects, possibly due to the low power in the individual studies. In another meta-analysis (Jo Wilson et al. 2003), the findings of mainstream programs (i.e., not culturally tailored for minority youth) for juvenile delinquency were synthesized to examine whether these programs had different outcomes on their subsequent antisocial behavior, academic performance, peer relations, and so on. Their results revealed the positive and equal effect of these programs for both minority and majority youth. Hence, more research is needed to examine the differences in treatment outcome between minority and majority youth.

This study is an attempt at further investigating potential predictors of differences in response to MST treatment in a homogenous treatment situation (i.e., the Norwegian Child Welfare System). In sum, we explored subgroups of youth classified by their response to MST for each ultimate MST outcome over time in Norway and whether immigrant background, in addition to gender and age of the youth at intake, predicted belonging to the subgroups.

## Method

### Participants and Procedure

The data for this study originated in the program data collection, which is an integral part of the continuous quality improvement system for MST in Norway.^1^ The data collection was originally licensed by The Norwegian Data Protection Authority (DPA), but as the EU General Data Protection Regulation (GDPR) was implemented in 2018, licensure was no longer optional, and was replaced by written consent from the families. Since the original purpose of collecting data was quality assurance of the treatment model, the relevant Norwegian authorities (The Regional Committee for Medical and Health Research Ethics (REK) and The Norwegian Centre for Research Data (NSD)) were consulted to ensure the ethics and legality of using these data for inclusion in the current article.

All MST cases admitted into the Norwegian Center for Child Behavioral Development (NUBU) database system and completed on or after May 10, 2012 and who also completed 18-month follow up MST before December 31, 2019, were included in this study. Data came from 1674 adolescents and their families referred to MST treatment by the municipal Child Welfare Services for serious and persistent antisocial behavior. Mean age of the adolescents at the start of MST was 14.55 years (*SD* = 1.58). 60.7% of the adolescents were boys. Additionally, 87.3% of adolescents were non-immigrants, while 12.7% were either 1^st^ or 2^nd^ generation immigrants. Adolescents with immigrant background mostly had an Asian (36.6%), European (30.0%) or African (13.6%) origin.

Data were collected by the MST team leaders who were in charge of assembling and entering the data into an internet database. At intake (T1), data reflected referral information from the Child Welfare Services and from the MST team’s initial assessment. At discharge (T2), information from families and from the MST team’s interaction with the family were used in the assessment of the youth. At 6-, 12- and 18- month follow-ups (T3, T4 and T5, respectively), the assessments by the caregivers were collected based on telephone interviews. To ensure valid and reliable data and minimize rater biases, supervisors and interviewers assessed outcomes according to detailed guidelines for scoring.

### Measures

Demographic information and immigrant status of the youth was created on the basis of the information provided through the Child Welfare Service referral documents and by the family. Youth born in Norway or abroad to two foreign-born parents, were categorized as immigrant, while children born to at least one Norwegian-born parent were categorized as nonimmigrant.

***Ultimate Outcomes.*** For the youth and their families who completed treatment (i.e., the treatment was not terminated due to placement out of home, or a lack of working alliance with the family, or for other reasons not related to the progress of treatment, such as illness/decease, or the family moving out of the team’s service area), the five ultimate outcomes of MST were assessed at all-time points (i.e., intake, discharge, and 6-, 12- and 18-month follow-ups): *(1) Living at home*: The youth was not placed out of home by the Child Welfare Services. *(2) Attending school/work:* The youth was attending school (was not truant) or vocational training or, if of the legally appropriate age to not attend school, had a paying job (at least half-time). *(3) Not in trouble with the law*: The youth did not receive any formalized, societal consequences as a result of crimes or misdemeanors. *(4) Abstaining from substance abuse:* The youth did not use any substances in a way that impaired daily functioning or led to other serious consequences. *(5) Abstaining from violence:* The youth did not use violence or threats of violence. Each of these outcomes were rated as *0* = No or *1* = Yes.

### Statistical Analyses

SPSS 21 was used for descriptive and multinomial regression analyses (MNR), and Mplus 7 (Muthén and Muthén 2012) was used for latent class growth analyses (LCGA). To explore heterogeneous trajectories regarding youths’ responses to MST, LCGA, containing a fixed number of five time points (T1–T5), were conducted separately for each outcome variable. LCGA as a longitudinal data analytic method identifies distinct latent subgroups based on changes in a variable over time (Nagin and Odgers, 2010). The LCGA, in which no variance within latent classes, only between classes, is allowed, is the least computationally complex model.

Data collected at intake (T1), discharge (T2), and 6- (T3), 12- (T4), and 18- (T5) month follow-ups after discharge were used in these analyses. Both models included an intercept and a linear slope. The loadings from the latent slope factor to each of the measures were 0, 5, 11, 17 and 23, reflecting the time interval measured by months (i.e., initial assessment at the intake, second assessment five months after intake at the discharge,^2^ third assessment 6-month after discharge, fourth assessment one year after discharge, and fifth assessment 18-month after discharge).

For each outcome, the two-class model was first specified, which was then used as a comparison for models of increasing class numbers until the best fitting model was identified.^3^ The model specification was stopped if a class with no participants emerged. The final best-fitting models were determined based on using goodness of fit criteria: a low Akaike’s information criterion (AIC), a low Bayesian information criterion (BIC) and sample size-adjusted Bayesian information criterion (SSBIC), and a significant improvement in model fit based on the Bootstrapped Likelihood Ratio Test (BLRT) (Nagin and Odgers 2010). Moreover, parsimony, high entropy, high average posterior probability of belonging to each cluster, and a distinctive course for each trajectory, were also considered in justification and interpretability of the trajectories (Jung and Wickrama 2008; Nylund et al. 2007). Maximum likelihood estimation with robust standard errors (MLR) was used to deal with missing data and correct for non-normality in the variables (Muthén and Muthén 2012).

After determination of the number of classes on each ultimate outcome with LGCA without the predictors, a new class categorical variable was created and saved based on most likely class membership for each individual. Finally, to identify predictors of trajectories generated through the LCGA, a series of MNRs were conducted in SPSS, with class membership as outcome, and immigrant status, age at intake and gender as predictors. The ‘*sustained improvement*’ group was specified as the reference group for comparison with the other groups. In the MNR analyses, if the overall likelihood ratio test was significant, we proceeded with the analysis by examining the significance of a predictor in distinguishing between specific group memberships. The MNR analyses produced odds ratios and 95% confidence intervals for the associations of each predictor variable adjusted for the influence of all the other variables entered in the model. The model fit was evaluated using the model chi-square and pseudo-R^2^. In addition, we adjusted p-value^4^ to test for significance to avoid inflated likelihood of error due to multiple comparisons.

## Results

### Descriptives

Table 1 presents the demographic variables for the overall sample as well as the percentage of ‘Yes’ responses for each ultimate outcome over time.

**Table 1:** Descriptives for the Total Sample (N = 1674)

	Descriptives	Missing data (%)
Age Mean (SD)	14.55(1.58)	-
% Male Gender	60.7	-
% Immigrant	12.7	-
*Living at home (% Yes)*		
Intake	92.2	-
Discharge	99.4	-
6-month follow-up	93.4	8.1
12-month follow-up	90.0	6.4
18-month follow-up	88.5	0.05
*Attending school/work (% Yes)*		
Intake	30.5	-
Discharge	88.9	-
6-month follow-up	84.5	8.1
12-month follow-up	82.8	6.4
18-month follow-up	81.8	0.05
*Not in trouble with the law (% Yes)*		
Intake	46.7	-
Discharge	97.7	-
6-month follow-up	95.1	8.1
12-month follow-up	94.0	6.4
18-month follow-up	93.7	0.05
*Abstaining from substance abuse (% Yes)*		
Intake	54.4	-
Discharge	95.9	0.05
6-month follow-up	91.7	8.2
12-month follow-up	90.5	6.4
18-month follow-up	90.2	0.05
*Abstaining from violence (% Yes)*		
Intake	26.2	-
Discharge	96.5	-
6-month follow-up	90.4	8.1
12-month follow-up	91.0	6.4
18-month follow-up	91.2	0.05

### Missing Data and Attrition

Missing values on the outcome variables included in the models ranged from 0.1%—8.2% (see Table 1). There was no missing data on main predictors of the latent classes. In order to examine attrition, missing assessment time points were examined with particular focus on monotone patterns of missingness (e.g. assessed at intake, but missing at all later time points). There was no missing at intake (T1) and discharge (T2), except only one missing response for abstaining from substance abuse variable at T2. Only one case had one missing among all variables at T5 (18-month follow up) assessment, making monotone missing patterns virtually nonexistent. At T3 (6-month follow up) there were 8.1% (N = 135) missing for all variables except abstaining from substance abuse variable with 8.2% (N = 137) missing. Lastly at T4 (12-month follow up), for all of the variables, there were 6.4% (N = 107) missing.

### LCGA Analyses: Determination of the Number of Latent Classes

***Living at Home.*** Fit indices for the two-class solution were AIC = 3463.86, BIC = 3475.09, SSBIC = 3490.97, entropy = 0.882, and BLRT < 0.001. Compared to the two-class solution, despite lower entropy, AIC, BIC, SSBIC, and BLRT showed better fit for three-class solution, but not for four-class solution. 5-class solution was still run, but was discarded due to a group with zero participant. Hence, both in terms of interpretability and parsimony, three class solution, i.e., a *deterioration after discharge* trajectory (6.1%, n = 103), a *gradual deterioration* trajectory (9.7%, n = 163), and a *sustained improvement* trajectory (84.1%, n = 1408), was accepted.

***Attending School/Work.*** Fit indices for the two-class solution were AIC = 7083.29, BIC = 7110.41, SSBIC = 7094.52, entropy = 0.841, and BLRT < 0.001. Compared to the two-class solution, despite lower entropy, AIC, BIC, and SSBIC showed better fit for both 3- and 4- class solutions, with significant BLRT. 5-class solution was still run, but was discarded due to a group with zero participant. Even though fit indices suggested the four-class solution compared to the three-class solution, in terms of interpretability and parsimony we explored more before deciding. In the four-class solution, the *sustained improvement* group was split into two groups with intercepts very close to each other, and parallel slopes. Hence, three class solution, i.e., a *gradual improvement* trajectory (28.9%, n = 484), a *gradual deterioration* trajectory (3.1%, n = 51), and a *sustained improvement* trajectory (68.0%, n = 1139), was selected.

***Not in Trouble with the Law.*** Fit indices for the two-class solution were AIC = 4685.23, BIC = 4712.35, SSBIC = 4696.46, entropy = 0.836, and BLRT < 0.001. A 3-class solution, despite higher entropy, showed a worse fit with higher AIC, BIC, and SSBIC, and significant BLRT, compared to the two-class solution. Since 3-class solution showed worse fit indices as well as significant BLRT, two class solution, i.e., a *gradual improvement* trajectory (12.7%, n = 213), and a *sustained improvement* trajectory (87.3%, n = 1461), was accepted.

***Abstaining from Substance Abuse.*** Fit indices for the two-class solution were AIC = 5358.40, BIC = 5385.51, SSBIC = 5369.63, entropy = 0.877, and BLRT < 0.001. Both 3- and 4- class solutions, despite lower entropy, AIC, BIC, and SSBIC showed better fit with significant BLRT, compared to the two-class solution. A 4-class solution, showed a better fit with better entropy, lower AIC, SSBIC, and significant BLRT, compared to the three-class solution. 5-class solution was run, but was discarded due to a group with zero participant. Hence, 4-class solution, i.e., a *gradual improvement* trajectory (12.2%, n = 204), a *gradual deterioration* trajectory (3.3%, n = 56), a *deterioration after 6-month* (2.1%, n = 35), and a *sustained improvement* trajectory (82.4%, n = 1379), was accepted.

***Abstaining from Violence.*** Fit indices for the two-class solution were AIC = 5185.72, BIC = 5212.83, SSBIC = 5196.95, entropy = 0.867, and BLRT < 0.001. A 3-class solution, despite lower entropy, showed a better fit with lower AIC, BIC, and SSBIC, and significant BLRT, compared to the two-class solution. Since 4-class solution showed worse fit indices as well as significant BLRT, three class solution, i.e., a *gradual improvement* trajectory (11.7%, n = 196), a *gradual deterioration* trajectory (6.0%, n = 102), and a *sustained improvement* trajectory (82.2%, n = 1376), was accepted.

**Fig. 1:**
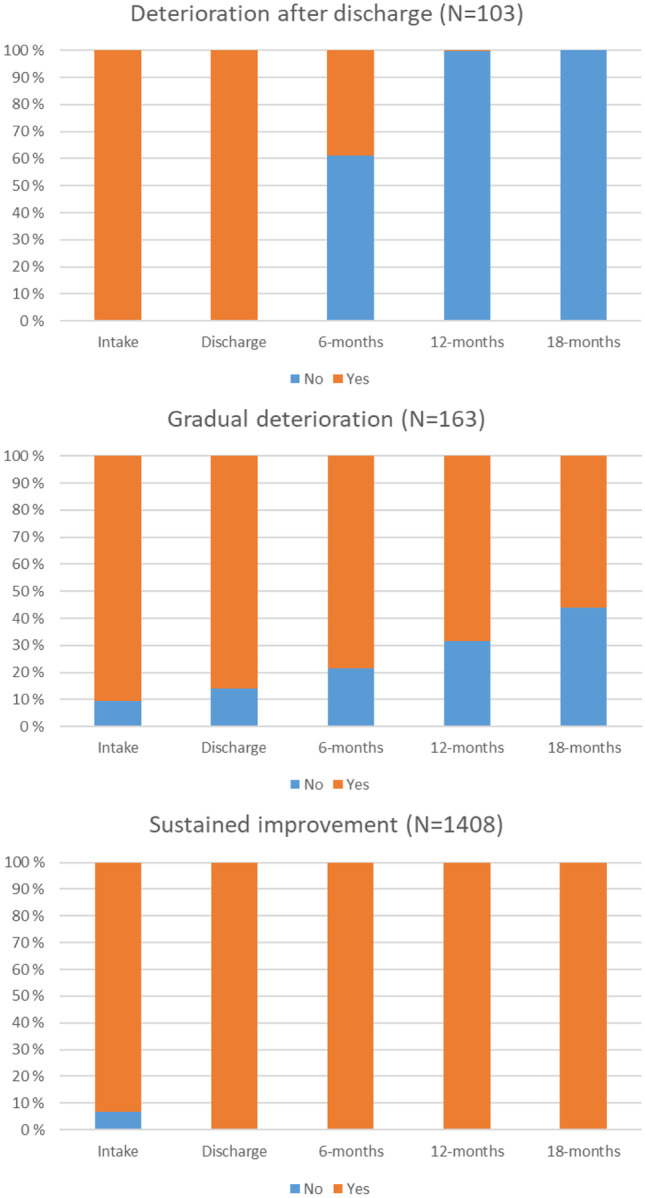
For ‘Living at home’ outcome, proportion of subjects reporting ‘yes’ or ‘no’ at each time point for each of the classes

**Fig. 2:**
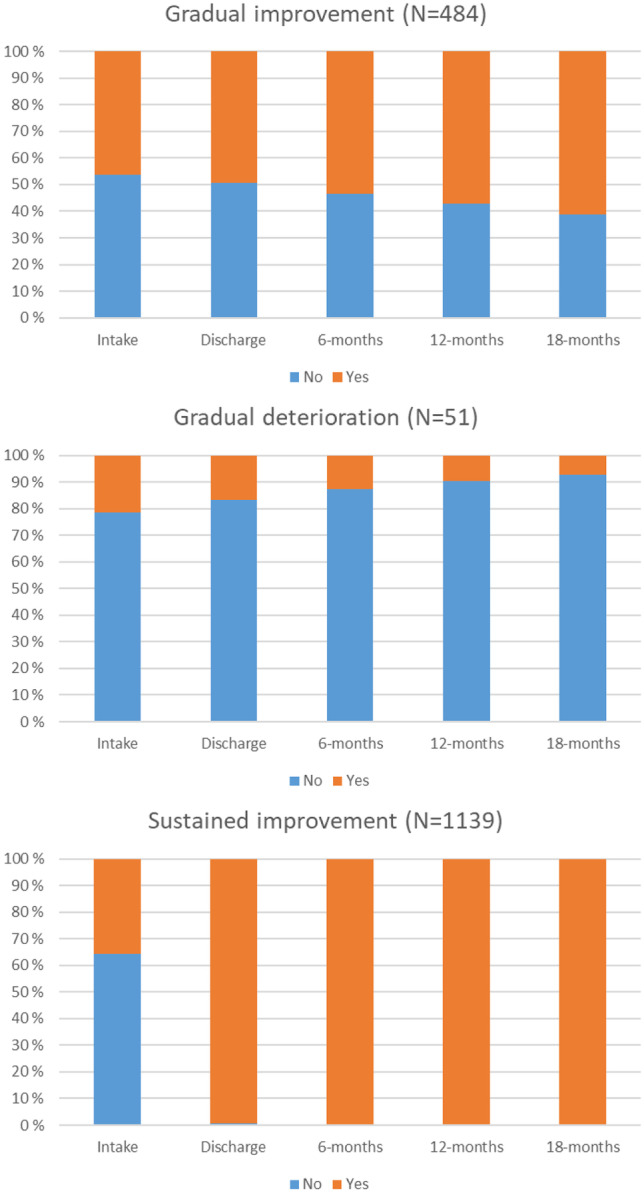
For ‘Attending school/work’ outcome, proportion of subjects reporting ‘yes’ or ‘no’ at each time point for each of the classes

**Fig. 3:**
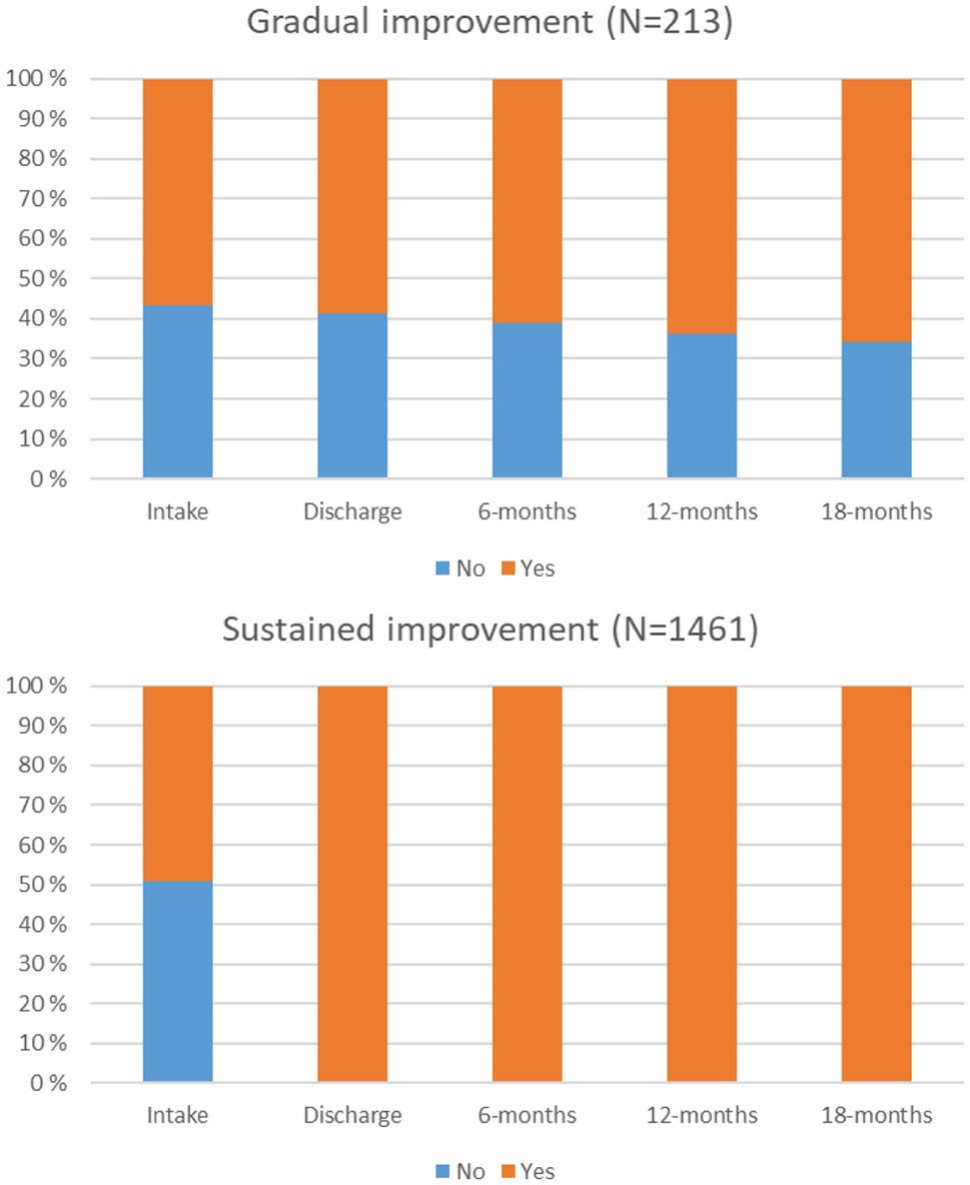
For ‘Not in trouble with the law’ outcome, proportion of subjects reporting ‘yes’ or ‘no’ at each time point for each of the classes

**Fig. 4:**
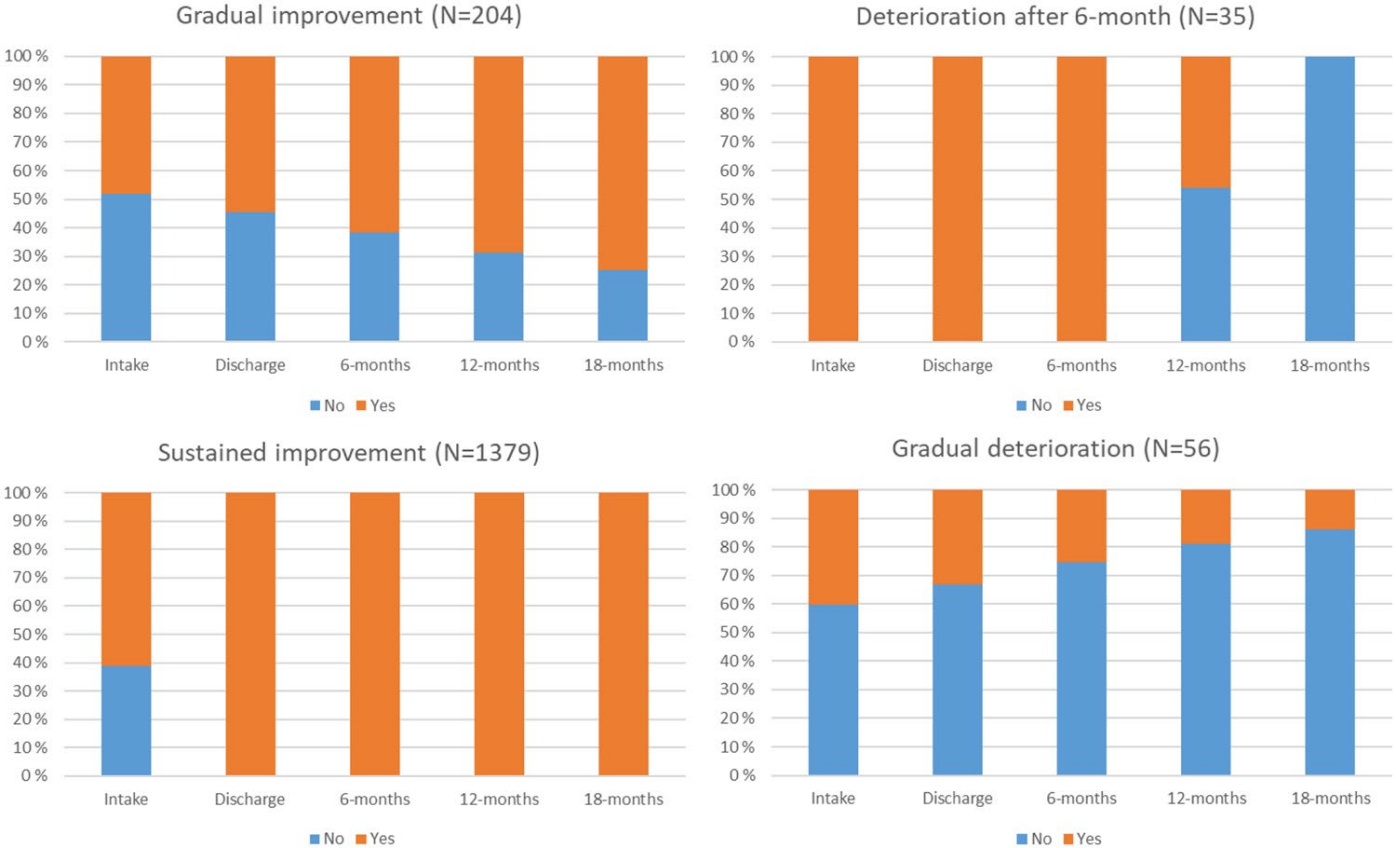
For ‘Abstaining from substance abuse’ outcome, proportion of subjects reporting ‘yes’ or ‘no’ at each time point for each of the classes

**Fig. 5:**
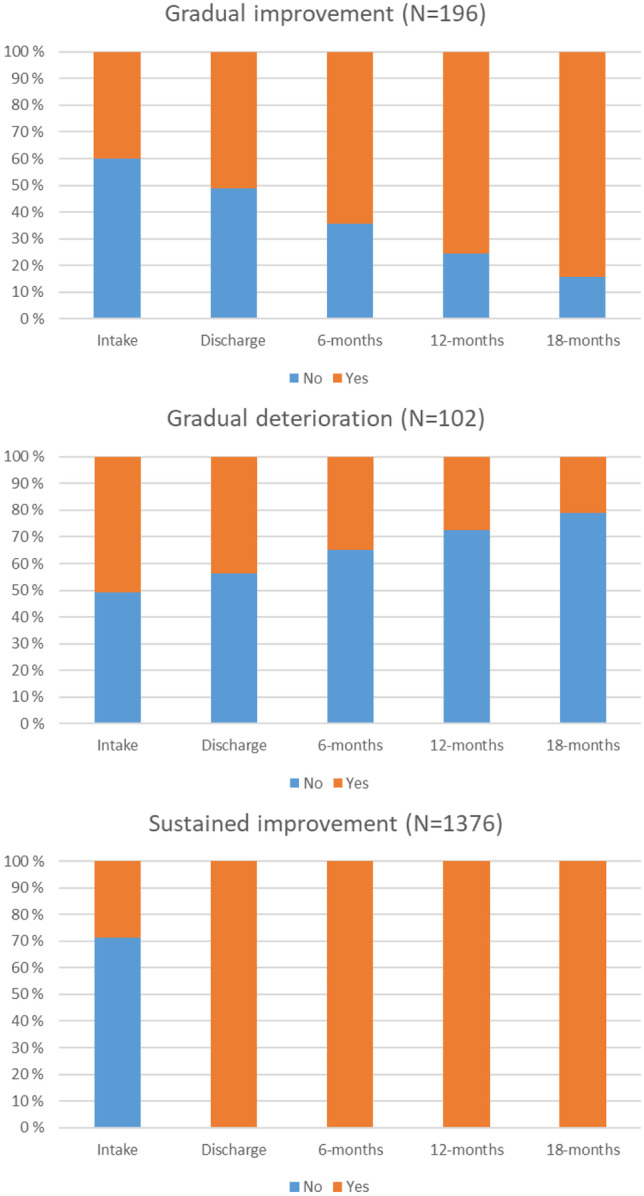
For ‘Abstaining from violence’ outcome, proportion of subjects reporting ‘yes’ or ‘no’ at each time point for each of the classes

Table 2 displays the model fit indices and entropy for each class solution in LCGA for each ultimate outcome. The estimated trajectories were graphically displayed for each outcome in Figs. 1a-5a (see Appendix A). Overall, for each ultimate outcome there was a trajectory (*sustained improvement*) with the highest percentage displaying the positive change after MST treatment at discharge; however, some of the trajectories also showed a poor treatment response, as some classes deteriorated slowly and others displayed a drastic deterioration over time. Overall, 800 youth (47.8%) belonged to the *sustained improvement* group across all outcomes.

**Table 2:** Model Fit Statistics for the LCGA Models (N = *1674)*

	AIC	BIC	SSBIC	Entropy	BLRT
*Living at home*					
M1	4155.22	4166.07	4159.71	-	-
M2	3463.86	3475.09	3490.97	0.882	< 0.001
**M3**	**3411.16**	**3454.54**	**3429.12**	**0.818**	** < 0.001**
M4	3409.80	3469.45	3434.51	0.825	= 0.050
*Attending school/work*					
M1	8658.44	8669.28	8662.93	-	-
M2	7083.29	7110.41	7094.52	0.841	< 0.001
**M3**	**7057.40**	**7100.79**	**7075.37**	**0.808**	** < 0.001**
M4	7045.40	7105.05	7070.11	0.859	< 0.001
*Not in trouble with the law*					
M1	5697.59	5708.44	5702.08	-	-
**M2**	**4685.23**	**4712.35**	**4696.46**	**0.836**	** < 0.001**
M3	4688.32	4731.71	4706.29	0.853	= 0.429
*Abstaining from substance abuse*					
M1	6519.87	6530.72	6524.36	-	-
M2	5358.40	5385.51	5369.63	0.877	< 0.001
M3	5347.66	5391.04	5365.63	0.843	< 0.001
**M4**	**5334.61**	**5394.27**	**5359.32**	**0.864**	** < 0.001**
*Abstaining from violence*					
M1	6809.98	6819.83	6813.47	-	-
M2	5185.72	5212.83	5196.95	0.867	< 0.001
**M3**	**5163.39**	**5206.77**	**5181.36**	**0.809**	** < 0.001**
M4	5169.39	5229.04	5194.10	0.847	= 1.00

*M* = model, *AIC* = Akaike information criterion, *BIC* = Bayesian information criterion, *SSBIC* = Sample size adjusted BIC, *BLRT* = Bootstrap likelihood ratio test. Best-fitting models were displayed in bold.

Figure 1,2,3,4,5 show the proportion of participants reporting ‘yes’ or ‘no’ at each time point for each of the classes, separately for each ultimate outcome. The descriptives of each latent class for each ultimate outcome are also presented in Table 3.

**Table 3:** Descriptives of the Latent Classes

	C1	C2	C3	C4
*Living at home*	*Deterioration after discharge* *(N = 103)*	*Gradual deterioration* *(N = 163)*	*Sustained improvement* *(N = 1408)*	
Mean(SD) Age	14.88(1.31)	14.69(1.53)	14.51(1.60)	
% Male	54.4	59.5	61.3	
% Immigrant	19.4	12.9	12.2	
*Attending school/work*	*Gradual improvement* *(N = 484)*	*Gradual deterioration* *(N = 51)*	*Sustained improvement* *(N = 1139)*	
Mean(SD) Age	14.95(1.38)	15.06(1.36)	14.36(1.63)	
% Male	59.3	56.9	61.5	
% Immigrant	11.4	3.9	13.7	
*Not in trouble with the law*	*Gradual improvement* *(N = 213)*	*Sustained improvement* *(N = 1461)*		
Mean(SD) Age	14.79(1.38)	14.51(1.60)		
% Male	76.1	58.5		
% Immigrant	17.4	12.0		
*Abstaining from substance abuse*	*Gradual improvement* *(N = 204)*	*Deterioration after 6-month* *(N = 35)*	*Sustained improvement* *(N = 1379)*	*Gradual deterioration* *(N = 56)*
Mean(SD) Age	15.30(1.17)	14.43(1.27)	14.41(1.61)	15.34(1.33)
% Male	67.2	65.7	59.0	75.0
% Immigrant	14.2	17.1	12.5	8.9
*Abstaining from violence*	*Gradual improvement* *(N = 196)*	*Gradual deterioration* *(N = 102)*	*Sustained improvement* *(N = 1376)*	
Mean(SD) Age	14.17(1.53)	13.69(1.89)	14.67(1.53)	
% Male	76.0	75.5	57.4	
% Immigrant	13.3	11.8	12.7	

*C* = class

### MNR Analyses: Predictors of the Latent Classes

To identify predictors of trajectories generated through the LCGA, multinomial regression analyses (MNRs) were conducted separately for each outcome, with class membership as outcome, and immigrant status, age at intake and gender as predictors. In each MNR, the *sustained improvement* group was specified as the reference group for comparison with the other groups. Table 4 presents the associations between the immigrant status, gender and age in predicting cluster membership.^5^

**Table 4:** Multinomial Regression relating Sustained Improvement Group to Immigrant Background and Demographic Factors

*Adjusted OR (95% CI) for Sustained Improvement group*^*a*^* relative to*											
Living at home	Deterioration after discharge *(N = 103)*			Gradual deterioration (N = 163)							
*Predictors*	*OR*	*p*	*CI*	*OR*	*p*	*CI*				$\underline {LR {\chi}^{{2}}}_{{2}}$	*p*
Age	1.172	0.026	[1.019–1.347]	1.078	0.169	[0.969–1.200]				6.69	0.035
Male Gender	0.785	0.241	[0.523–1.177]	0.953	0.778	[0.683–1.330]				1.40	0.498
Nonimmigrant	0.566	0.030	[0.338–0.948]	0.933	0.781	[0.574–1.517]				4.26	0.119
Attending school/work	Gradual improvement (N = 484)			Gradual deterioration (N = 51)							
*Predictors*	*OR*	*p*	*CI*	*OR*	*p*	*CI*				$\underline {LR {\chi}^{{2}}}_{{2}}$	*p*
Age	**1.298**	**0.000**	[1.203–1.400]	**1.371**	**0.003**	[1.116–1.684]				55.30	0.000
Male Gender	0.998	0.983	[0.800–1.244]	0.920	0.775	[0.520–1.628]				0.82	0.960
Nonimmigrant	1.229	0.224	[0.881–1.713]	3.848	0.064	[0.925–16.009]				6.20	0.045
Not in trouble with the law^b^	Gradual improvement (N = 213)										
*Predictors*	*OR*	*p*								$\underline {\chi 2(3)}$	*p*
Age	**1.157**	**0.003**								39.07	0.000
Female Gender	**2.371**	**0.000**									
Immigrant	0.652	0.033									
Abstaining from substance abuse	Gradual improvement (N = 204)			Deterioration after 6-month (N = 35)			Gradual deterioration (N = 56)				
*Predictors*	*OR*	*p*	*CI*	*OR*	*p*	*CI*	*OR*	*p*	*CI*	$\underline {LR {\chi}^{{2}}}_{{2}}$	*p*
Age	**1.575**	**0.000**	[1.403–1.767]	1.023	0.835	[0.826–1.266]	**1.617**	**0.000**	[1.314–1.989]	87.84	0.000
Male Gender	**1.616**	**0.003**	[1.176–2.221]	1.339	0.423	[0.656–2.733]	**2.398**	**0.006**	[1.291–4.451]	16.56	0.001
Nonimmigrant	0.856	0.484	[0.554–1.323]	0.697	0.428	[0.285–1.703]	1.462	0.428	[0.571–3.739]	1.85	0.605
Abstaining from violence	Gradual improvement (N = 196)			Gradual deterioration (N = 102)							
*Predictors*	*OR*	*p*	*CI*	*OR*	*p*	*CI*				$\underline {LR {\chi}^{{2}}}_{{2}}$	*p*
Age	**0.847**	**0.000**	[0.773–0.928]	**0.724**	**0.000**	[0.646–0.812]				37.31	0.000
Male Gender	**2.206**	**0.000**	[1.557–3.123]	**1.954**	**0.005**	[1.218–3.133]				27.90	0.000
Nonimmigrant	0.984	0.945	[0.629–1.540]	1.158	0.650	[0.614–2.183]				0.28	0.893

*OR* = odds ratio, *CI* = confidence interval *LR*- likelihood ratio test chi square. ^a^ (*N* = 1408 for Living at home, *N* = 1139 for Attending school/work, *N* = 1461 for Not in trouble with the law, *N* = 1379 for Abstaining from substance abuse, *N* = 1376 for Abstaining from violence). ^b^ Logistic regression results, Significant predictors after adjusting p value.(p < .017) were shown in bold.

***Living at Home.*** According to the likelihood ratio tests, no predictor variable revealed significant relation to the outcome classification. Hence, none of the predictors could differentiate the *sustained improvement* group from the *gradual deterioration* and *deterioration after discharge* groups.

***Attending School/Work.*** According to the likelihood ratio tests, only age (likelihood ratio, χ2_2_ = 55.30, *p* < 0.001) revealed significant relation to the outcome classification. Younger youth were more likely to belong to the *sustained improvement* group, compared to the *gradual deterioration* (OR = 1.37, *p* = 0.003) and *gradual improvement* groups (OR = 1.30, *p* < 0.001).

***Not in Trouble with the Law.*** Since two groups emerged as the result of LCGA, a logistic regression was performed to ascertain the effects of immigrant status, gender and age on the likelihood that participants belong to the *sustained improvement* group. The logistic regression model was statistically significant, χ2_3_ = 39.08, *p* < 0.001. The model explained 4.3% (Nagelkerke R^2^) of the variance and correctly classified 87.3% of cases. Girls were more likely to belong to the *sustained improvement* group (OR = 2.37, *p* < 0.001), compared to the *gradual improvement* group. Increasing age, on the other hand, was associated with a decreased likelihood of belonging to the *sustained improvement* group (OR = 1.18, *p* = 0.003).

***Abstaining from Substance Abuse.*** According to the likelihood ratio tests, both age (likelihood ratio, χ2_2_ = 87.84, *p* < 0.001) and gender (likelihood ratio, χ2_2_ = 16.56, *p* = 0.001) revealed significant relation to the outcome classification. Younger youth were more likely to belong to the *sustained improvement* group, compared to the *gradual deterioration* (OR = 1.62, *p* < 0.001) and *gradual improvement* groups (OR = 1.58, *p* < 0.001). Moreover, girls were more likely to belong to the *sustained improvement* group, compared to the *gradual deterioration* and *gradual improvement* groups (OR = 2.40, *p* = 0.006, and OR = 1.62, *p* = 0.003, respectively). None of the predictors could differentiate the *sustained improvement* group from the *deterioration after 6-month* one. MNR was repeated by changing the reference group to *deterioration after 6-month* to further examine its differentiation from the other clusters. Compared to the *deterioration after 6-month* ones, those in the *gradual deterioration* and *gradual improvement* groups had lower odds of having older youth (OR = 1.58, *p* = 0.002, and OR = 1.54, *p* < 0.001, respectively).

***Abstaining from Violence.*** According to the likelihood ratio tests, both age (likelihood ratio, χ2_2_ = 37.31, *p* < 0.001) and gender (likelihood ratio, χ2_2_ = 27.90, *p* < 0.001) revealed significant relation to the outcome classification. Older youth were more likely to belong to the *sustained improvement* group, compared to the *gradual deterioration* (OR = 0.72, *p* < 0.001) and gradual improvement groups (OR = 0.85, *p* < 0.001). Moreover, girls were more likely to belong to the *sustained improvement* group, compared to the *gradual deterioration* and *gradual improvement* groups (OR = 1.95, *p* = 0.005, and OR = 2.21, *p* < 0.001, respectively).

## Discussion

In this study, extant research on Multisystemic Therapy (MST) was enriched by examining and identifying the subgroups of youth who responded differently to MST in terms of ultimate MST outcomes (i.e., living at home, attending school/work, not in trouble with the law, abstaining from substance abuse, and abstaining from violence). We further investigated whether the immigrant background, age at intake and gender predicted belonging to the subgroups that were identified.

Our results showed that there were two to four subgroups of youth for different MST outcomes. For each outcome, one large subgroup (the *sustained improvement* group), ranging from 68% to 84% of the overall sample, maintained the positive status or did not deteriorate after discharge as well as over time. Overall, approximately half of the youth (47.8%) belonged to the *sustained improvement* group across all outcomes, hence showing stable improvement for all MST outcomes. There were also two main groups that emerged for almost of all of the outcomes, a *gradual improvement* group (except for the ‘living at home’ outcome) and a *gradual deterioration* group (except for the ‘not in trouble with the law’ outcome) with the youth ranging approximately from 12% to 29%, and from 3% to 10% of the overall sample, respectively. Lastly, there were two small groups with deterioration emerged after discharge for ‘living at home’ outcome (i.e., a group in which more than half of the youth at 6-month follow-up, and all of them at the subsequent measurements, were placed out of home by the Child Welfare Services), and after 6-month for ‘abstaining from substance abuse’ outcome (i.e., a group in which more than half of the youth at 12-month follow-up, and all of them at 18-month follow-up, used substances in a way that impaired daily functioning or led to other serious consequences). These deterioration subgroups consisted of approximately 6% and 2% of the overall sample, respectively. Hence, the overall results indicated a high and sustained degree of improvement across the ultimate outcomes for the vast majority of the youths. However, even though a large group of the youth showed a trajectory with sustained improvement over time, there was still variation in the groups emerged with improvement and deterioration trajectories for various outcomes.

For all outcomes, except ‘living at home’, the next largest subgroup emerged was the *gradual improvement* one. This finding may point to a delayed treatment effect and this is in line with one of the main MST treatment principles. This principle emphasizes generalization of treatment interventions, so the family will be able to maintain and also potentially improve outcomes after treatment has ended. This may also reflect systemic ripple effects of treatment, generating increased contextual reinforcement of positive behaviors. Taken together, the *sustained improvement* and *gradual improvement* groups leave only a small proportion of the youths in groups showing deterioration after treatment. For the ‘living at home’ group, on the other hand, there was no *gradual improvement* group. This is due to the fact that nearly all youths were living at home at the beginning of treatment. The remaining fraction was moving home from a child welfare institution at the beginning of treatment. Nevertheless, a relatively larger proportion of the youths, approximately 16%, displayed some deteriorating pattern for this outcome compared to the other outcomes.

Following the identification of the subgroups, we further explored whether immigrant status, age at intake and gender predicted these various trajectories. Immigrant status did not differentiate the subgroups for any of the outcomes. We expected variation between immigrant and nonimmigrant youth due to a range of factors such as overall socioeconomic level, trust in and skepticism towards child welfare services (e.g., Tembo et al. 2020), language barriers for minority youths, in addition to cultural differences that families are embedded in. The absence of immigrant status predicting membership of any particular class in all of the outcomes, however, may indicate that MST worked just as well for immigrant youth as for non-immigrant youth, as MST is a culturally competent intervention (Brondino et al.^ 1997^). This may possibly reflect the flexibility in tailoring interventions according to the needs of each specific youth and family, which is a fundamental principle of MST. A recent qualitative study revealed that when MST therapists consider cultural differences and acknowledge them sensitively, and act as a cultural broker especially between youth and family, immigrant youth and families engage and change more during MST treatment, hence resulting in successful MST effect (Fox et al. 2017). Our finding is also consistent with the results of a meta-analysis demonstrating the effectiveness of mainstream service programs for various outcomes equally for ethnic minority and majority juvenile delinquents (Jo Wilson et al. 2003), even though the use of different programs and outcomes prevents a direct comparison with our results. It is, however, worth to mention that immigrant youth in our study may be a heterogeneous group, with diverse cultural and socioeconomic backgrounds. However, insufficient power in our study due to the small numbers per immigrant generation status or origin did not allow us to further examine differences among immigrant youth group. So, we should be aware of the fact that comparing a non-immigrant, native group of youth with a mix of various immigrant groups may lead to over-interpretation of the differences, and in addition may conceal large differences amongst immigrants (Anderson and Mayes 2010; Stevens and Vollebergh 2008).

Age at intake, on the other hand, was a significant predictor of belonging to the subgroups for all of the ultimate outcomes except ‘living at home’ outcome. Except for the ‘abstaining from violence’ outcome, older youth were less likely to belong to the *sustained improvement* trajectory. This is in line with the results of the meta-analysis showing that MST is more effective with younger youth (Van der Stouwe et al. 2014). In our study, we may also infer that while younger youth were more likely to show sustained improvement, older youth displayed a relatively poorer improvement across most of the ultimate outcomes and this may inform the way MST should be delivered. First, it underscores the need for early intervention. A prolonged history of serious behavior problems increases the risk for severe marginalization processes, increased affiliation with negative peers, and loss of positive developmental experiences on the various social arenas – thus also generating a greater risk of entering into a detrimental trajectory during adult life. MST emphasizes the parents as main drivers of change, whereas for older youth, there may be a need for increased work with youth involvement in therapy, and for more individual work with the youth.

On the other hand, older youth had a greater probability of belonging to the *sustained improvement* trajectory for the ‘abstaining from violence’ outcome. This may be a reflection of increased neurological maturation, associated with improved executive control of attention and self-regulation capacity – which may play a greater role regarding violence than for the other outcomes (Gardner et al. 2008; Spear 2000). However, we do not have any data to examine this, hence future studies may explore this. Also, we cannot rule out the fact that some of the youths in the dataset belong to the ‘late onset’ developmental trajectory (Moffitt and Caspi 2001), where problem behavior spontaneously decreases as the youth gets older. Moreover, our data is limited, as this variable covers a range of phenomena, such as not distinguishing between physical violence, and threats of physical violence. This may confound potential systematic changes with age. So, future studies are welcomed to investigate this in detail.

While there was no difference for boys and girls in predicting group membership for ‘living at home’ and ‘attending school/work’ outcomes, girls, on the other hand, were more likely to belong to the *sustained improvement* group for the other three outcomes (i.e., not in trouble with the law, abstaining from substance abuse, and abstaining from violence). It is interesting to note that these are the three outcomes which are more likely to be related to externalizing behaviours, and in the literature several findings suggest that girls tend to display lower levels of behavioral problems than boys (e.g., Bongers et al. 2004; Moffitt and Caspi 2001; Ogden and Hagen 2009). Since our study is the first exploring various subgroups of ultimate outcomes and the role of gender in predicting these trajectories over time, we do not have any literature to compare our findings. Moreover, as gender differences in MST outcomes have not been examined as a central topic in MST efficacy (Ogden and Hagen 2009), our finding is in line with the Norwegian RCT follow-up study (Ogden and Hagen 2006) which demonstrated gender as a significant predictor for some MST outcome areas and when reported by some informants.

Some limitations to this current study should be mentioned. In this explorative study, we examined a limited number of demographic predictors; and we were not able to investigate various individual (e.g., social competence, aggression), contextual (e.g., parental factors, peer characteristics, family characteristics) or implementation-related (e.g., the site and/or the therapist characteristics, alliance with therapist) factors subject to change in predicting various trajectories. Examining the role of these changeable factors may provide valuable information on how to improve treatment response by addressing and manipulating these factors early in treatment (Mertens et al. 2017). Moreover, we did not examine the potential interaction among our predictors such as the interaction of age and gender. Even though our sample size was relatively large, due to the LCGA analyses and emergence of small groups, there was still a lack of power in our dataset to detect interaction effects for any of our small groups. The ultimate goals were evaluated as dichotomous variables, with an either-or response; hence, variation and range in these outcomes (e.g., a youth who breaks the law many times versus another one who does once) cannot be captured. With regard to the data in this study, it should also be mentioned once again that the data for this study was not originally collected as research data, but as the program data collection registered by different MST teams, as a part of the continuous quality improvement system for MST in Norway. The program data were also collected from different data sources for the different time points. That is, while data at intake and discharge involved MST team assessment, follow-up data involved parents’ assessments. Moreover, the ultimate outcomes were measured at all-time points only for MST completers. Hence, it is important to emphasize that the potential generalizability of the study pertains to completers (i.e., 88.4% of the cases ending treatment in MST Norway during the period in question), and not to all youth and families going through MST treatment. Non-completers were not included as they were not asked to submit follow-up information due to ethical considerations. However, additional attrition analyses between completers and non-completers revealed that even though there was not a significant gender difference between these two groups, there was a tendency for a reduced completion rate for older youths, and immigrant families tended to complete treatment at a lower rate than non-immigrant families. Hence, our results should be interpreted with caution in the light of these limitations of our sample’s representativeness. Last but not least, the generalizability of our findings is limited by a lower prevalence of antisocial disorders in the Nordic countries compared to other countries worldwide (Skogen and Torvik 2013).

Despite these limitations, this study still adds valuable information to our knowledge about how youth respond to MST. Our results suggest that not every youth responds similarly to MST, and there is heterogeneity in their response over time as well as the predictors of the subgroups of youth across different outcomes. More studies examining heterogeneity will possibly help us to identify factors that may be targeted in order to better tailor the MST interventions for youth with serious problems.

## Electronic Supplementary Material

Below is the link to the electronic supplementary material.

Supplementary file1 (DOCX 787 kb)

## Footnotes

1 Different from international program data, Norwegian MST data include ultimate outcomes regarding abstaining from violence, which has not been included in other countries, and abstaining from substance abuse, which has been an optional outcome outside of Norway only since 2018. In addition, internationally, these outcomes have been mainly measured only at discharge, but not at admission or follow-ups. In the current study, hence, we were able to explore the subgroups of youth regarding their responses to MST for each ultimate MST outcome over time in Norway.

2 In the current sample, the time between intake and discharge was on average approximately 5 months, in accordance with the general length of an MST treatment.

3 One-class solution, which had worse model fit compared to two-class solution for all of the outcomes, was also performed and reported on in Table 2.

4 We calculated this using Bonferroni's method which gave us our new threshold of significance (p < .017).

5 We also graphically displayed the proportion of participants reporting ‘yes’ at each time point for significant predictors across each of the classes, separately for each ultimate outcome (see Appendix B).
