# Supplementary material for: Identification of Multisystemic Therapy (MST) Subgroups with Distinct Trajectories on Ultimate Outcomes in Norway
Source: Res Child Adolesc Psychopathol. 2021 Jan 6;49(4):429–42. doi: 10.1007/s10802-020-00735-3 (PMC7943514; doi:10.1007/s10802-020-00735-3)
Supplement: Supplementary file 1 — Supplementary file1 (DOCX 787 kb) [file 10802_2020_735_MOESM1_ESM.docx]

**Appendix A**


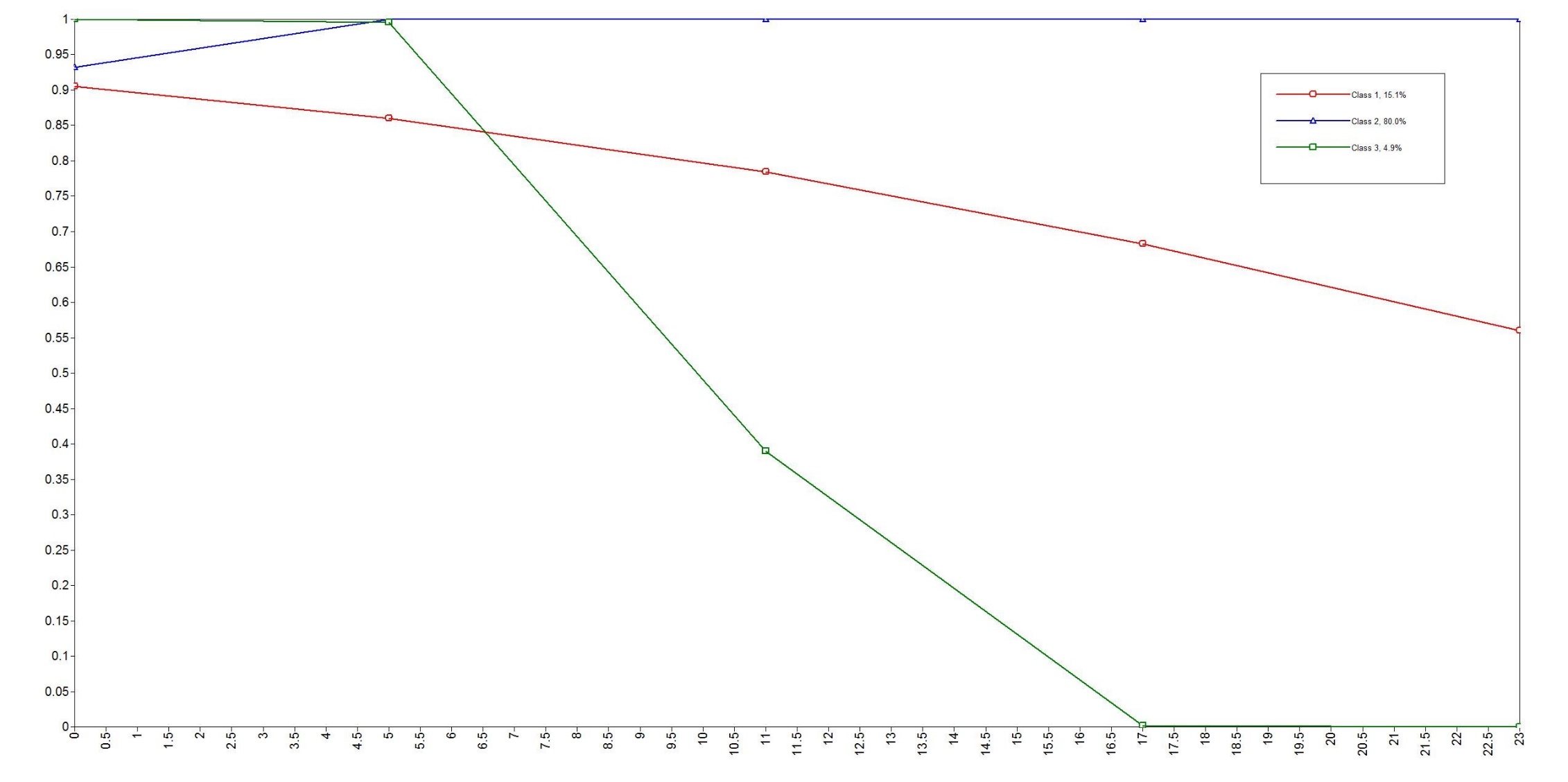


*Figure 1a.* The estimated trajectories for ‘Living at home’ outcome. Class 1= Gradual deterioration, Class 2= Sustained improvement, and Class 3= Deterioration after discharge.


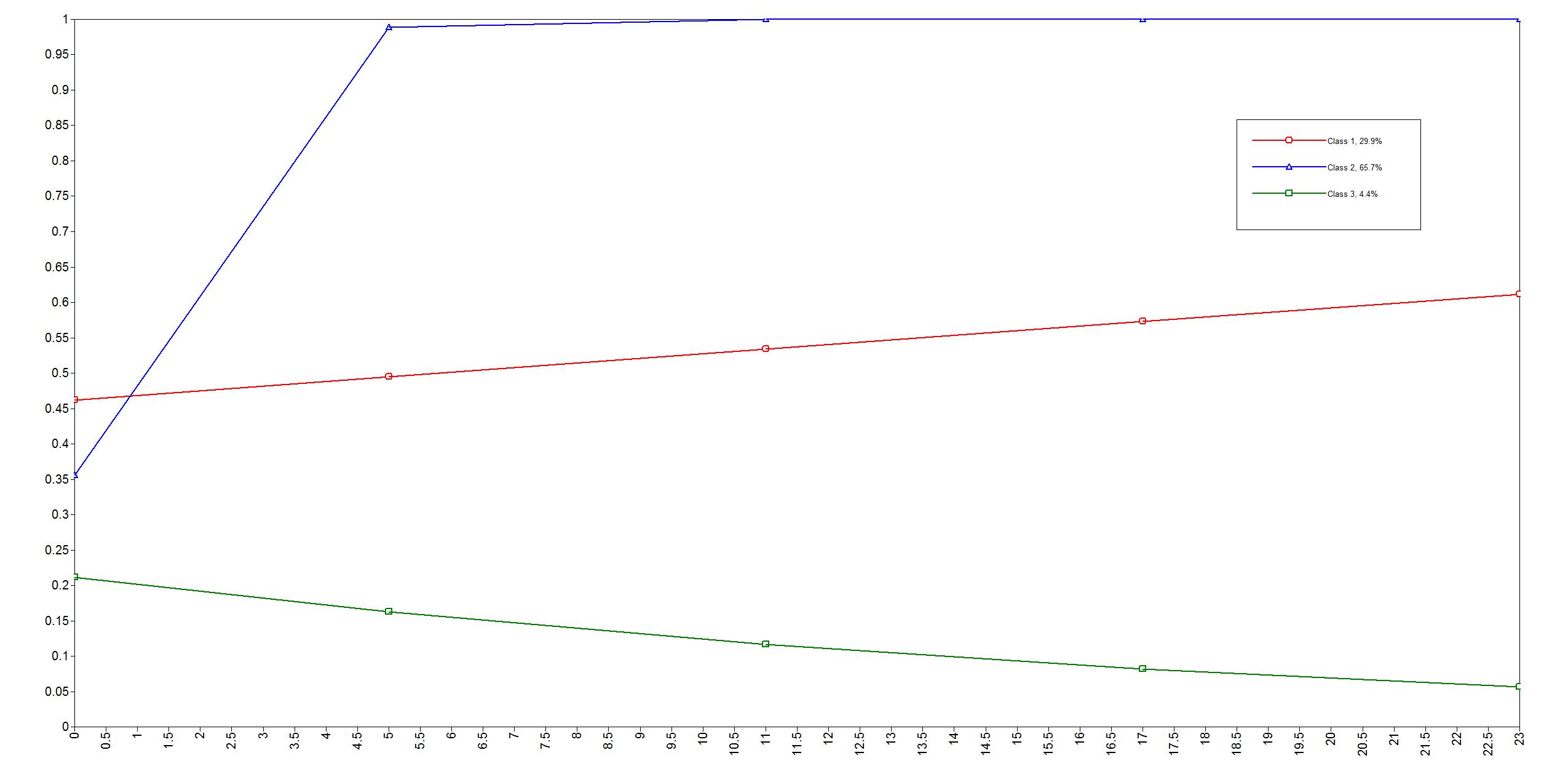


*Figure 2a.* The estimated trajectories for ‘Attending school/work’ outcome. Class 1= Gradual improvement, Class 2= Sustained improvement, and Class 3= Gradual deterioration.


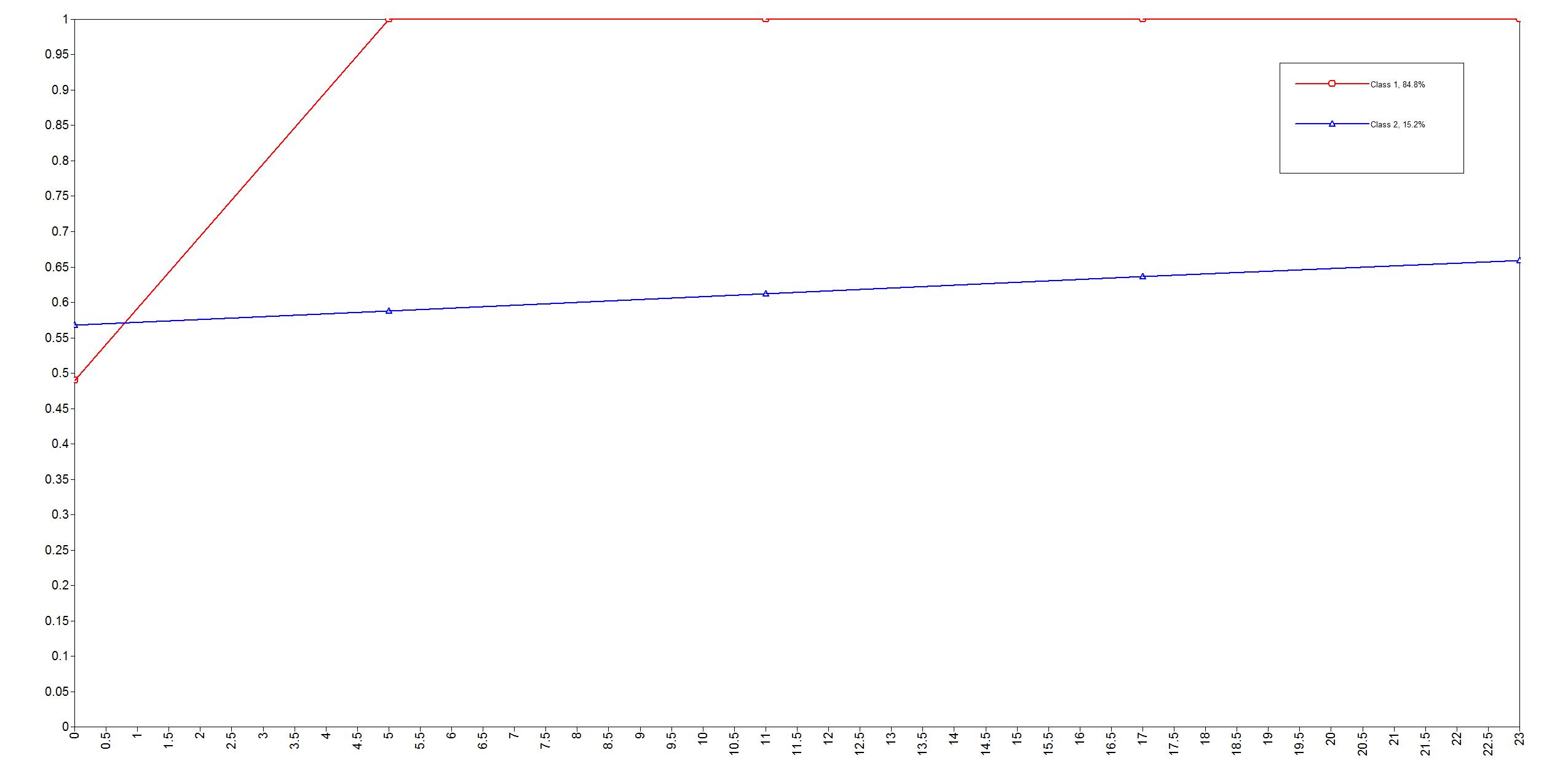


*Figure 3a.* The estimated trajectories for ‘Not in trouble with the law’ outcome. Class 1= Sustained improvement and Class 2= Gradual improvement.


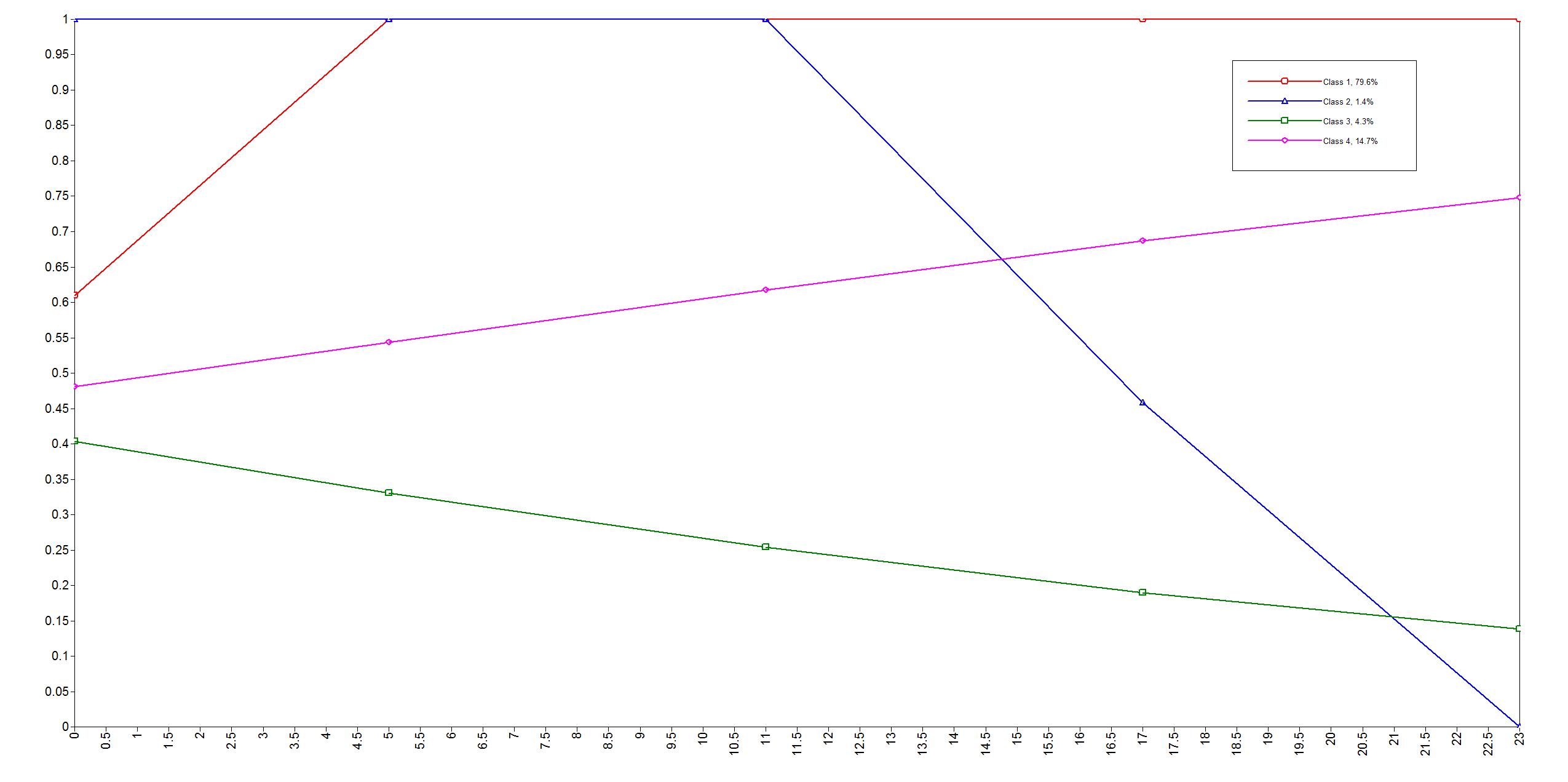


*Figure 4a.* The estimated trajectories for ‘Abstaining from substance abuse’ outcome. Class 1= Sustained improvement, Class 2= Deterioration after 6-month, Class 3= Gradual deterioration, and Class 4= Gradual improvement.


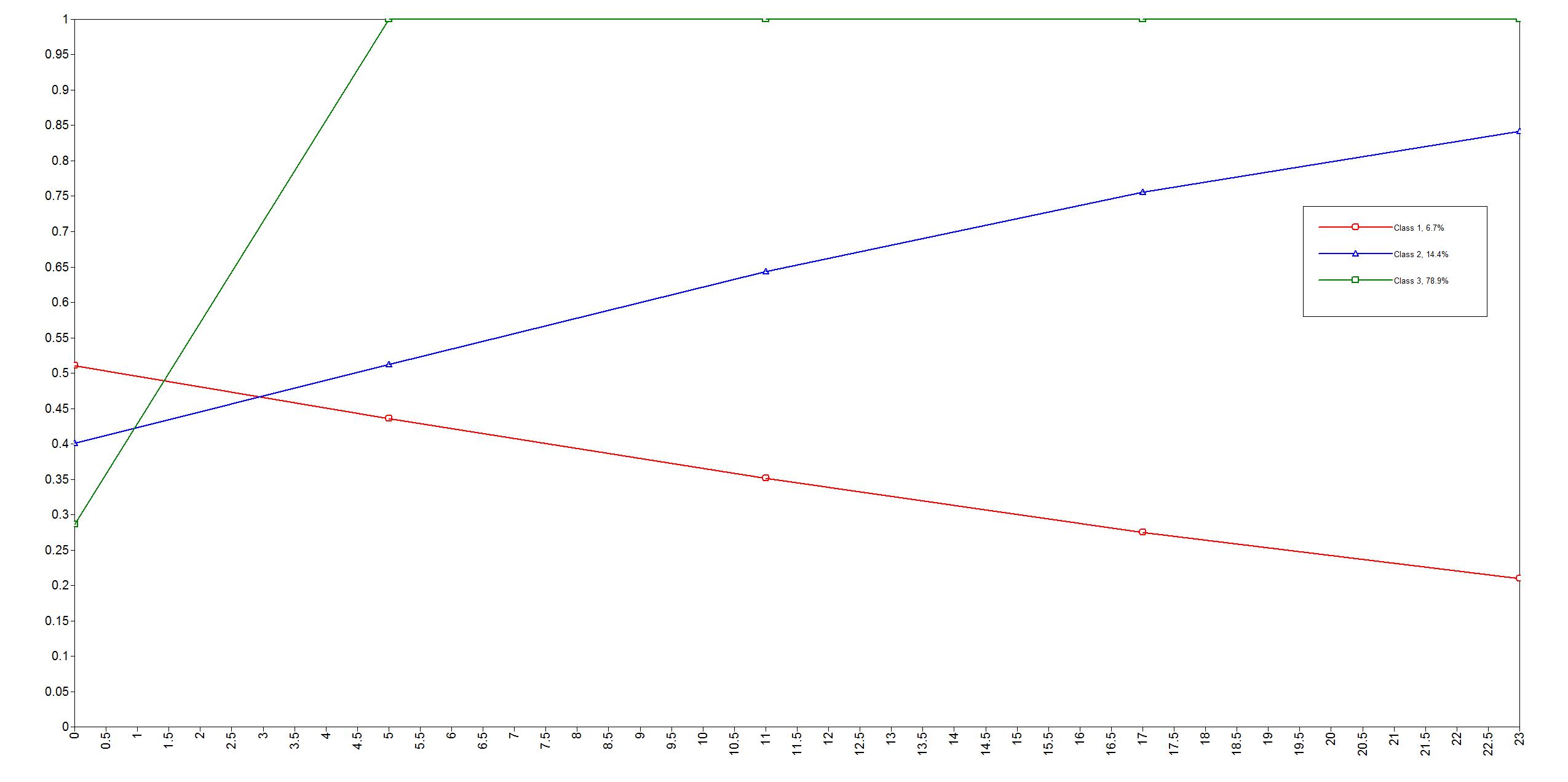


*Figure 5a.* The estimated trajectories for ‘Abstaining from violence’ outcome. Class 1= Gradual deterioration, Class 2= Gradual improvement, and Class 3= Sustained improvement.

**Appendix B**


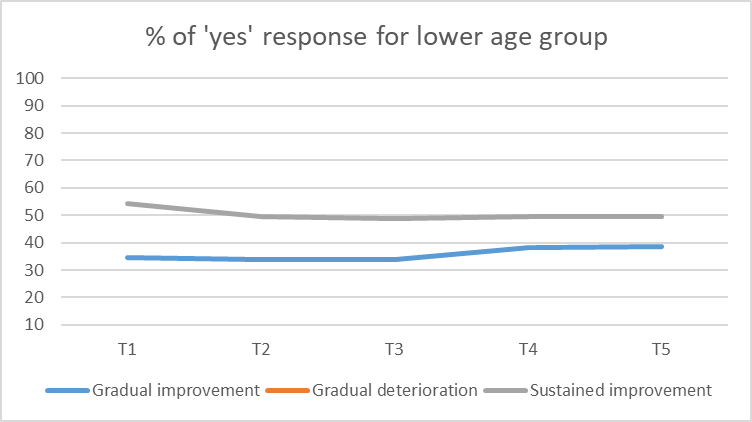


*Figure 1b.* The observed trajectories of significant predictors for ‘Attending school/work’ outcome.

*Notes.* The age variable was dichotomized as “lower age group” referring to the participants below the mean age, and “higher age group” referring to the participants above the mean age.

*
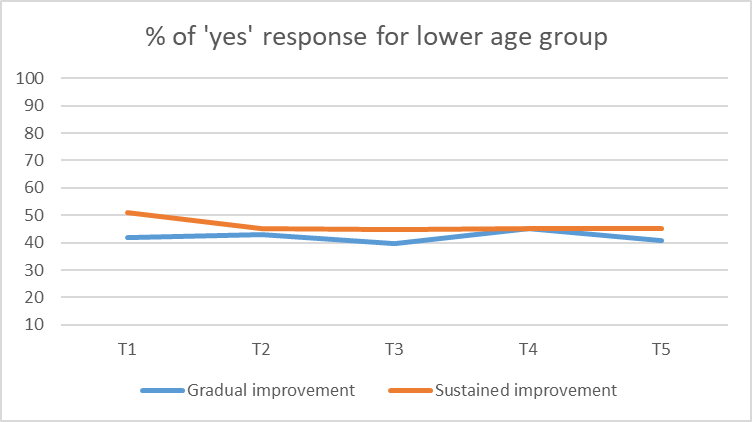
*

*
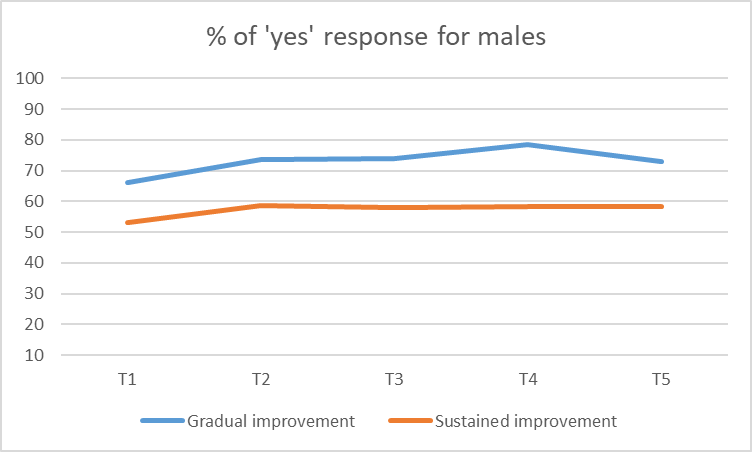
*

*Figure 2b.* The observed trajectories of significant predictors for ‘Not in trouble with the law’ outcome.


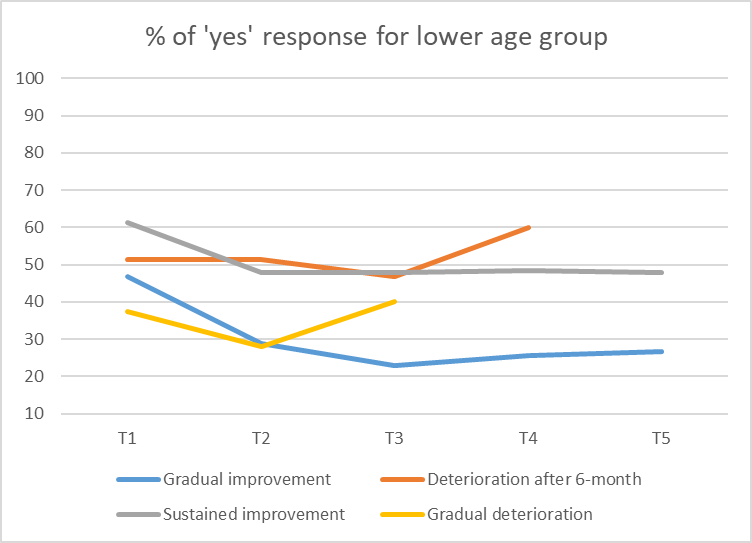


*
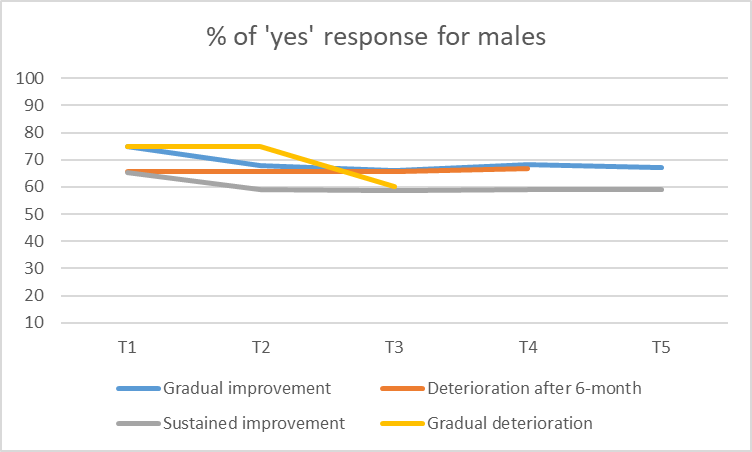
*

*Figure 3b.* The observed trajectories of significant predictors for ‘Abstaining from substance abuse’ outcome.


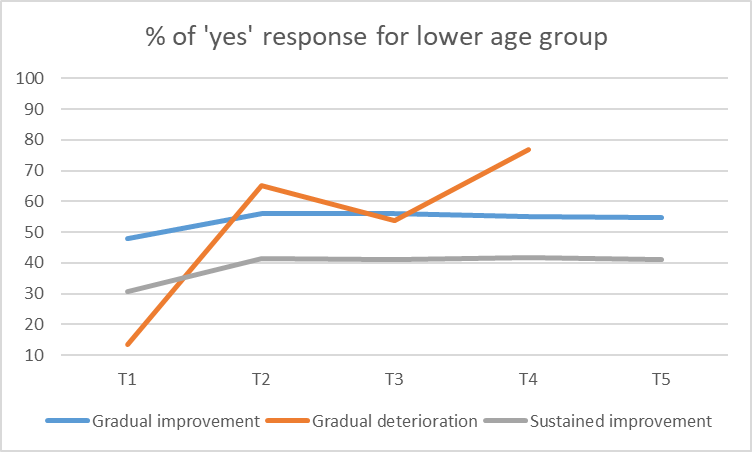


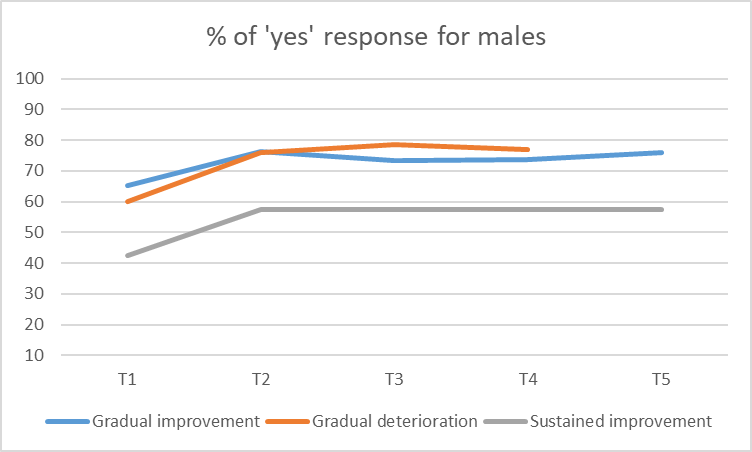


*Figure 4b.* The observed trajectories of significant predictors for ‘Abstaining from violence’ outcome.
